# Methionine Sulfoxide Reductases Are Essential for Virulence of *Salmonella* Typhimurium

**DOI:** 10.1371/journal.pone.0026974

**Published:** 2011-11-02

**Authors:** Luisa A. Denkel, Sarah A. Horst, Syed Fazle Rouf, Vera Kitowski, Oliver M. Böhm, Mikael Rhen, Timo Jäger, Franz-Christoph Bange

**Affiliations:** 1 Department of Medical Microbiology and Hospital Epidemiology, Medical School Hannover, Hannover, Germany; 2 Molecular Links Sachsen-Anhalt Gesellschaft mit beschränkter Haftung, Magdeburg, Germany; 3 Department of Microbiology, Tumor and Cell Biology, Karolinska Institute, Stockholm, Sweden; Laurentian University, Canada

## Abstract

Production of reactive oxygen species represents a fundamental innate defense against microbes in a diversity of host organisms. Oxidative stress, amongst others, converts peptidyl and free methionine to a mixture of methionine-*S*- (Met-*S*-SO) and methionine-*R*-sulfoxides (Met-*R*-SO). To cope with such oxidative damage, methionine sulfoxide reductases MsrA and MsrB are known to reduce MetSOs, the former being specific for the *S*-form and the latter being specific for the *R*-form. However, at present the role of methionine sulfoxide reductases in the pathogenesis of intracellular bacterial pathogens has not been fully detailed. Here we show that deletion of *msrA* in the facultative intracellular pathogen *Salmonella* (*S.*) *enterica* serovar Typhimurium increased susceptibility to exogenous H_2_O_2_, and reduced bacterial replication inside activated macrophages, and in mice. In contrast, a Δ*msrB* mutant showed the wild type phenotype. Recombinant MsrA was active against free and peptidyl Met-*S*-SO, whereas recombinant MsrB was only weakly active and specific for peptidyl Met-*R*-SO. This raised the question of whether an additional Met-*R*-SO reductase could play a role in the oxidative stress response of *S*. Typhimurium. MsrC is a methionine sulfoxide reductase previously shown to be specific for free Met-*R*-SO in *Escherichia (E.) coli*. We tested a Δ*msrC* single mutant and a Δ*msrB*Δ*msrC* double mutant under various stress conditions, and found that MsrC is essential for survival of *S.* Typhimurium following exposure to H_2_O_2,_ as well as for growth in macrophages, and in mice. Hence, this study demonstrates that all three methionine sulfoxide reductases, MsrA, MsrB and MsrC, facilitate growth of a canonical intracellular pathogen during infection. Interestingly MsrC is specific for the repair of free methionine sulfoxide, pointing to an important role of this pathway in the oxidative stress response of *Salmonella* Typhimurium.

## Introduction

*Salmonella* affects humans and animals causing a variety of diseases including gastroenteritis, septicemia, and typhoid fever. *Salmonella* is a facultative intracellular pathogen. Inside macrophages, the pathogen is exposed to oxidative stress [1]. Individuals with chronic granulomatous disease, who suffer from a genetic defect of the phagocyte NADPH oxidase gene, show a severely impaired production of reactive oxygen intermediates (ROI) due to the lack of superoxide anion O_2_^-^, and are more susceptible to infections with *Salmonella* [2]. Likewise, susceptibility to *Salmonella* is increased in macrophages from mice with diminished NADPH oxidase activity [3]. Pathogens counteract oxidative stress by detoxifying ROI and repairing damage caused by ROI. For detoxification *Salmonella* possesses two classes of enzymes, catalases and peroxiredoxin-type peroxidases (peroxiredoxins).Three members of the catalase family, KatG, KatE, and KatN, and three members of the peroxiredoxin family, AhpC, TsaA, and Tpx are present in *Salmonella,* pointing to the multiple enzymes evolved in *Salmonella* for this route [4]–[6].

Despite detoxification, repair is an essential part of oxidative stress response in most bacteria. Exposure to ROI particularly affects sulfur residues, due to their high susceptibility to oxidation [7]. Sulfur containing amino acids such as methionine can be easily repaired to their native form by specific enzymes [7]. It has been suggested that reduction of methionine not only repairs damage afflicted by oxidative defense of the host, but even provides an advantage, as it could scavenge oxidants and thereby preventing other, less readily reversible reactions [8]. Methionine sulfoxide, whether in protein or as free amino acid, exists in two epimers, methionine-(*S*)-sulfoxide (Met-*S*-SO) and methionine-(*R*)-sulfoxide (Met-*R*-SO) [7]. Two sulfoxide reductases encoded by *msrA* and *msrB*, mediate reduction of Met-*S*-SO and Met-*R*-SO, respectively [7], [9]. In *E. coli* MsrA acts on both free and peptidyl Met-*S*-SO whereas MsrB acts on peptidyl Met-*R*-SO and shows little activity for free Met-*R*-SO [10]. Repair of free Met-*R*-SO has been shown to be mediated by a third methionine sulfoxide reductase encoded by *msrC* [10]–[12].

In bacteria such as *Mycobacterium* (*M.*) *smegmatis* [13], *Staphylococcus* (*S.*) *aureu*s [14], and *E. coli* [15] mutants of *msrA* were hypersensitive to ROI. In *Campylobacter* (*C.*) *jejuni* [16], and *Enterococcus* (*E*.) *faecalis* [17], mutants of *msrA* and *msrB* showed hypersensitivity towards ROI, and the effect was much more pronounced in the *msrA* mutant than in the *msrB* mutant. In *Helicobacter* (*H*.) *pylori* and *Neisseria (N.) gonorrhoeae msrA* and *msrB* are fused together. Deletion of the fusion protein increased sensitivity towards ROI in both species [18], [19]. However, none of these bacterial species are typical intracellular pathogens. In *Mycobacterium* (*M.*) *tuberculosis* deletion in either *msrA* or *msrB* did not affect resistance to ROI *in vitro* [20]. Thus at present the role of MsrA and MsrB for pathogenesis of intracellular pathogens has not been fully understood. There are even less data available on the role of MsrC in bacterial pathogenesis. Recently its ability to be stereo and size specific exclusively for the reduction of free Met-*R*-SO has been described in *S. aureus*, *E. coli*, *N. meningitides* and in *Saccharomyces* (*S.*) *cerevisiae* [10], [11], [21], [22]. However, except for *S. cerevisiae* a role of MsrC in oxidative response has not been reported in these organisms.

Here we generated single and double mutants of *msrA* and *msrB* and showed that *msrA* is essential for survival of *S.* Typhimurium exposed to exogenous H_2_O_2_, and growth of the pathogen in activated macrophages and in mice. In contrast, a Δ*msrB* mutant showed the wild type phenotype. Further experiments showed that MsrB has weak reductive activity towards peptidyl Met-*R*-SO and no activity towards free Met-*R*-SO. Repair of free Met-*R*-SO was carried out by MsrC. Δ*msrC* single and Δ*msrB*Δ*msrC* double mutants were more susceptible to exogenous H_2_O_2_, showed reduced growth in activated macrophages, and the Δ*msrB*Δ*msrC* double mutant was attenuated in mice. These results not only suggest that the methionine sulfoxide reductase pathway plays a key role in oxidative stress response of *S.* Typhimurium, but also indicates that the repair of free oxidized amino acids contributes significantly to resistance of the pathogen towards oxidative stress.

## Results

### *In vitro* growth and survival of Δ*msrA* and Δ*msrB* mutants following exposure to exogenous H_2_O_2_

**Figure 1:**
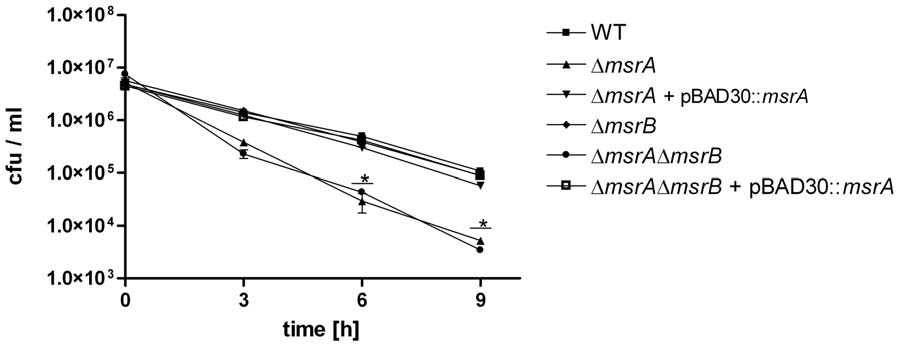
Susceptibility of *S.* Typhimurium Δ*msrA,* Δ*msrB*, Δ*msrA*Δ*msrB,* Δ*msrA* + pBAD30::*msrA,* and Δ*msrA*Δ*msrB*+ pBAD30::*msrA* towards exogenous H_2_O_2_. Wild type and mutant strains Δ*msrA*, Δ*msrB*, Δ*msrA*Δ*msrB,* Δ*msrA* + pBAD30::*msrA* and Δ*msrA*Δ*msrB* + pBAD30::*msrA* were exposed to 2 mM exogenous H_2_O_2_ in LB medium, and plated for colony forming units at the indicated time points. Analysis was done in duplicates, and is a representative of three independent experiments. Asterisks indicate *P*-values <0.05.

STM4408 was annotated as *msrA* on the chromosome of *S.* Typhimurium, with 89% identity (amino acids) to MsrA (AAC77176) of *E. coli*. STM1291 was annotated as *yeaA* on the chromosome of *S.* Typhimurium, with 86% identity (amino acids) to *yeaA* (AAC74848) of *E. coli* (*coli*BASE) [23]. Subsequently, *yeaA* of *E. coli* was renamed to *msrB* [24]. For this present study, we initially generated mutants in *S.* Typhimurium with deletions in *msrA* (STM4408), and *msrB* (STM1291), and double mutants with deletions in both genes. Neither mutant revealed any general growth defect under normal aerobic culture conditions in LB broth (Fig. S1). The impact of the mutations on the susceptibility of *S*. Typhimurium towards exogenous H_2_O_2_ was assessed *in vitro*. In the presence of 2 mM exogenous H_2_O_2_ the Δ*msrA* mutant was more susceptible than the wild type after 6 and 9 hours of exposure to H_2_O_2_ (Fig. 1). The wild type phenotype could be restored by expressing the wild type *msrA* on a plasmid under the control of its own promoter. In contrast, survival of the Δ*msrB* mutant was not affected in the presence of 2 mM H_2_O_2_ *in vitro* after 6 and 9 hours of exposure to H_2_O_2_. A Δ*msrA*Δ*msrB* mutant was constructed to test whether the double mutant has a stronger phenotype than the *msrA* single mutant in the presence of H_2_O_2_. The results shown in Figure 1 indicate that the phenotype of the single Δ*msrA* and the double Δ*msrA*Δ*msrB* mutant do not differ from each other. This is in line with the fact that the single *msrA* from wild type was sufficient to restore the phenotype of the Δ*msrA*Δ*msrB* double mutant (Fig. 1).

### Growth of Δ*msrA* and Δ*msrB* mutants inside activated macrophages

**Figure 2:**
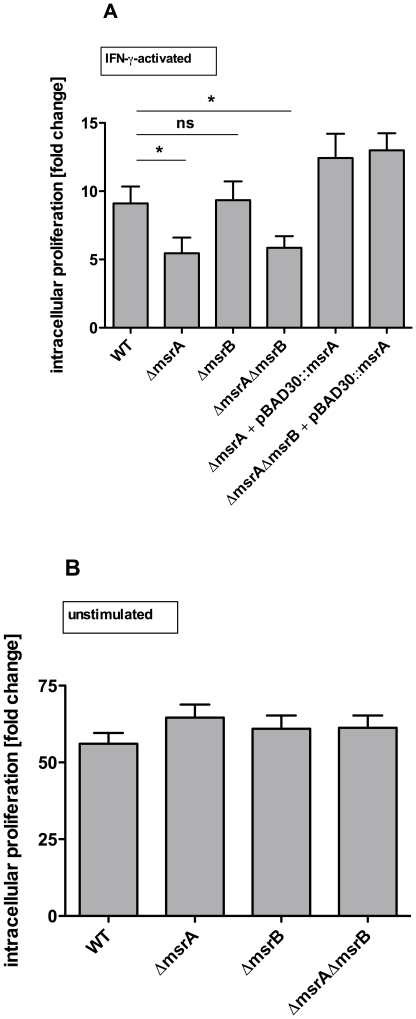
Intracellular proliferation of *S.* Typhimurium wild type, Δ*msrA*, Δ*msrB*, Δ*msrA*Δ*msrB* in RAW 264.7 cells. **A.** *S.* Typhimurium wild type, Δ*msrA*, Δ*msrB*, Δ*msrA*Δ*msrB,* Δ*msrA* + pBAD30::*msrA* and Δ*msrA*Δ*msrB* + pBAD30::*msrA* were tested in IFN-γ-activated RAW 264.7 cells**.** **B.** *S.* Typhimurium wild type, Δ*msrA*, Δ*msrB*, and Δ*msrA*Δ*msrB*, were tested in non-stimulated RAW 264.7 cells. Intracellular proliferation was determined in a gentamicin protection assay. Number of bacteria 16 h post infection was divided by the number of bacteria 2 h post infection. Results shown are the means ± standard error of the mean for three independent experiments, each in triplicate. *P-*values <0.05 were considered to be significant as indicated by asterisks. ns: not significant.

To elucidate the role of *msrA* and *msrB* during intracellular growth of *S*. Typhimurium, the murine cell-line RAW 264.7 was infected with wild type and mutant strains. Intracellular bacteria were enumerated 2 and 16 hours (h) post infection (p.i.) by plating for colony forming units. Proliferation was calculated by dividing the bacterial numbers 16 h p.i. by the number of bacteria 2 h p.i.. In IFN-γ-activated RAW 264.7 macrophages the single Δ*msrA* and the Δ*msrA*Δ*msrB* double mutant were attenuated to the same extent, while growth of the Δ*msrB* mutant was not affected (Fig. 2A). Complementation of the Δ*msrA*Δ*msrB* double mutant with the wild type *msrA* was sufficient to restore the wild type phenotype. Similar studies addressing the role of Msr in *E. faecalis*, *Actinobacillu*s (*A.*) *actinomycetemcomitans* and *M. smegmatis* used non-stimulated cells as controls [13], [17], [25]. Thus we tested the strains in non-stimulated RAW 264.7 cells, and found that neither the Δ*msrA* and Δ*msrB* single mutants, nor the Δ*msrA*Δ*msrB* double mutant were affected in their intracellular growth in comparison to the wild type (Fig. 2B).

### *In vivo* susceptibility of Δ*msrA* and Δ*msrB* mutants in Balb/cJ mice

To test whether the deletion of *msrA* or *msrB* affects virulence of *S.* Typhimurium in mice, the fitness of the mutants was directly compared to that of the wild type in a competition experiment [26]. For each mutant and time point groups of 5 female Balb/cJ mice were infected intraperitoneally with a mixture of two bacterial strains in a ratio of 1∶1 and bacterial burden in liver and spleen was enumerated 1 and 3 days after infection. Competitive indices (CI) were calculated as described elsewhere [26] and are given for bacterial loads of various strains in spleen and liver 1 day and 3 days after infection (Table 1 and 2). The Δ*msrA* mutant was significantly attenuated in the liver and spleen 1 and 3 days after infection. In contrast, the Δ*msrB* mutant was not attenuated in the mouse model of infection in the liver and spleen. The Δ*msrA*Δ*msrB* double mutant was also attenuated both in the liver and the spleen.

**Table 1:** Competitive indices (CI) of Δ*msrA*, Δ*msrB* and Δ*msrA*Δ*msrB* versus wild type on day 1 post infection.

Strains	CI liver 1 day p.i.	*P*-value*	CI spleen 1 day p.i.	*P*-value*
Δ*msrA* vs. WT	0.69±0.11	<0.05	0.81±0.10	<0.05
Δ*msrB* vs. WT	1.01±0.27	>0.05	0.93±0.27	>0.05
Δ*msrA*Δ*msrB* vs. WT	0.72±0.21	<0.05	0.62±0.11	<0.05

**Table 2:** Competitive indices (CI) of Δ*msrA*, Δ*msrB* and Δ*msrA*Δ*msrB* versus wild type on day 3 post infection.

Strains	CI liver 3 days p.i.	*P*-value*	CI spleen 3 days p.i.	*P*-value*
Δ*msrA* vs. WT	0.16±0.03	<0.05	0.24±0.07	<0.05
Δ*msrB* vs. WT	1.13±0.06	<0.05	1.12±0.09	>0.05
Δ*msrA*Δ*msrB* vs. WT	0.23±0.09	<0.05	0.13±0.05	<0.05

*P-values were calculated using the Wilcoxon Signed Rank Test to test whether the actual median differs significantly from 1 (theoretical median). CIs are shown as medians ± standard deviation.

### Biochemical characterization of MsrA and MsrB

**Figure 3:**
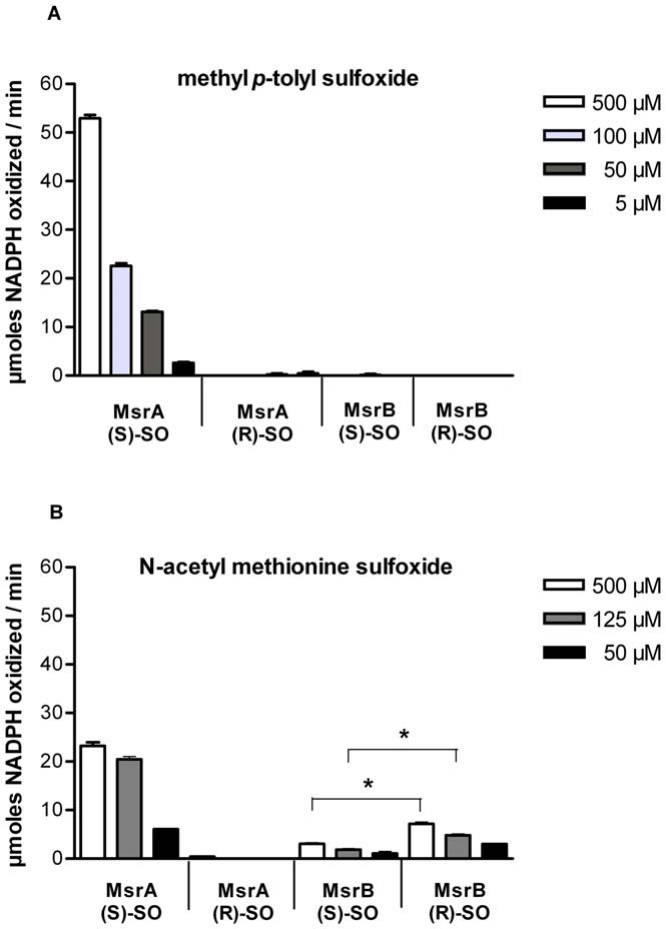
NADPH-linked reductase activity assay of recombinant MsrA and MsrB from *S.* Typhimurium. **A.** The graph shows the amount of oxidized NADPH per minute for the reduction of 500, 100, 50 and 5 µM methyl *p*-tolyl sulfoxide by MsrA and MsrB, respectively. **B.** The graph shows the amount of oxidized NADPH per minute for the reduction 500, 125 and 50 µM N-acetyl methionine sulfoxide by MsrA and MsrB, respectively. The decrease of absorbance at 340 nm due to NADPH oxidation was measured in the presence of the *S-* or *R*-isomer of methyl *p*-tolyl sulfoxide (A), and in the presence of the *S-* or *R-*isomere of N-acetyl methionine sulfoxide (B) using purified His-tagged MsrA or MsrB. Besides 10 µM purified MsrA or MsrB, the test system contained 10 µM TrxR, 10 µM TrxB, 450 µM NADPH, and various concentrations of *S*- or *R*-methyl *p*-tolyl sulfoxide (Sigma), and N-acetyl methionine sulfoxide (MOLISA), respectively. Reactions were filled up to a final volume of 500 µl with a buffer containing 50 mM HEPES and 1 mM EDTA (pH 7.4). Reactions were carried out at 25°C and started after 10 min pre-incubation by addition of the oxidized methionine derivatives.

To investigate the enzymatic activity of MsrA and MsrB we overexpressed both proteins in *E. coli* followed by purification of recombinant MsrA and MsrB. Enzymatic activities of MsrA and MsrB were tested initially with methyl *p*-tolyl sulfoxide, as both the *R*- and the *S*-form are commercially available. In a test system coupled to NADPH oxidation, thioredoxin (Trx) was used as an electron donor for Msr. MsrA was active against the *S-*epimer of methyl *p*-tolyl sulfoxide but not the *R*-epimer. MsrB displayed no activity against either epimer of methyl *p*-tolyl sulfoxide (Fig. 3A). N-acetyl methionine sulfoxide has been used as a surrogate for peptidyl MetSO [20]. We chemically synthesized N-acetyl Met-*R*-SO and N-acetyl Met-*S*-SO and tested activities of both MsrA and MsrB. MsrA and MsrB each were active against N-acetyl MetSO in a stereospecific manner, because MsrA reduced the *S*-form of N-acetyl MetSO, while MsrB reduced the *R*-form of N-acetyl MetSO (Fig. 3B). It seems that MsrB was active against N-acetyl Met-*S*-SO, too. But an NMR-analysis of this substrate showed, that N-acetyl Met-*S*-SO was not pure but contaminated with N-acetyl Met-*R*-SO. In fact, purity of N-acetyl Met-*S*-SO as determined by ^1^H-NMR was 80%. Thus the observed activity is due to this contamination.

### Utilization of methionine sulfoxide by a Δ*msrB* and a Δ*msrC* mutant

**Figure 4:**
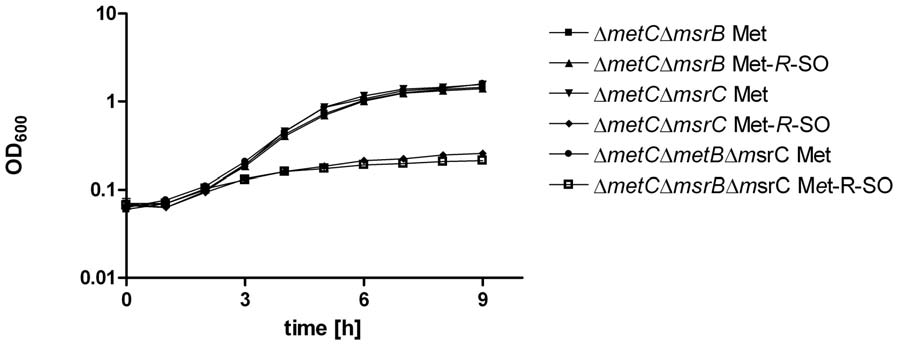
Growth of methionine auxotrophic mutants with deletions in *msrB* and *msrC* on methionine and free Met-*R*-SO. Bacteria were grown overnight in LB, harvested, washed and cultivated in M9 minimal medium supplemented with 13 µg ml^−1^methionine (Met) or methionine-*R*-sulfoxide (Met-*R*-SO) (MOLISA), respectively, started at an optical density (OD_600_) of 0.05. Growth of Δ*metC*Δ*msrB*, Δ*metC*Δ*msrC* and Δ*metC*Δ*msrB*Δ*msrC* was observed by measurement of OD_600_ every hour for nine hours of cultivation. Experiments were done in triplicates. Means are shown ± standard deviation.

The wild type phenotype of the mutant with a deletion in STM1291 (*msrB*) prompted us to perform a more detailed analysis of methionine sulfoxide reductases that specifically act on the Met-*R*-SO epimer. We included MsrC (STM1847) in our analysis, which had been shown to encode a methionine sulfoxide reductase that reduces free but not peptidyl Met-*R*-SO in *E. coli* [10]. The open reading frame STM1847 was annotated on the chromosome of *S.* Typhimurium, with 85% identity (amino acids) to fRMsr (AAC77176) of *E. coli* (*coli*BASE) [23]. Eventually fRMsr was named MsrC (EcoGene database at http://ecogene.org/ ), and this latter designation was used throughout this study. An auxotrophic mutant that is unable to synthesize methionine and deficient for methionine sulfoxide reductases fails to grow on MetSO, because MetSO cannot be used by acylate tRNA^met^ as a substrate [27]. Thus methionine auxotrophic mutants can be used to test the function of methionine sulfoxide reductases. Using the Δ*metC* background, a Δ*msrB* mutant was generated. The Δ*metC*Δ*msrB* double mutant could grow on Met-*R*-SO (Fig. 4), suggesting that lack of MsrB does not alter the ability of the mutant to reduce free Met-*R*-SO, which is in line with the enzyme activity data of MsrB. In contrast, the Δ*metC*Δ*msrC* double mutant and the Δ*metC*Δ*msrB*Δ*msrC* triple mutant were unable to grow on Met-*R*-SO. Taken together these results suggest that MsrC repairs free Met-*R*-SO in *S.* Typhimurium.

### *In vitro* growth and survival of a Δ*msrC* mutant following exposure to exogenous H_2_O_2_

**Figure 5:**
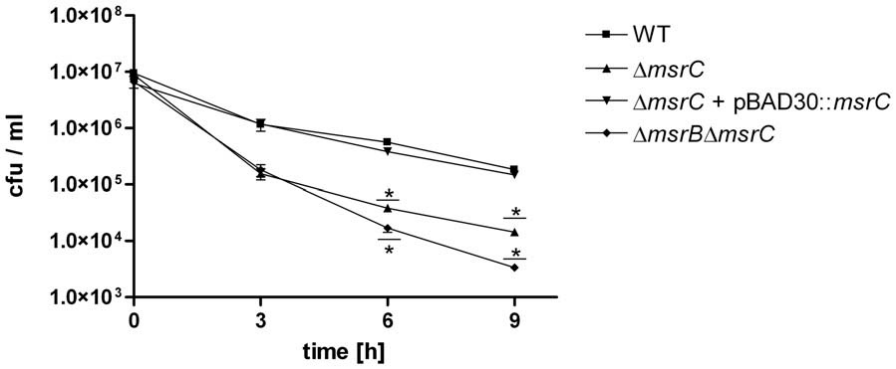
Susceptibility of *S.* Typhimurium Δ*msrC,* Δ*msrC* + pBAD30::*msrC* and Δ*msrB* Δ***msrC*** **towards exogenous H_2_O_2_.** Wild type and mutant strains Δ*msrC,* Δ*msrC* + pBAD30::*msrC* and Δ*msrB*Δ*msrC* were treated with 2 mM exogenous H_2_O_2_ in LB medium and plated for colony forming units at the indicated time points. The data shown are representatives and were performed at least three times with identical results. Experiments were done in duplicates and means are shown ± standard deviation. Asterisks indicate *P*-values <0.05.

**Figure 6:**
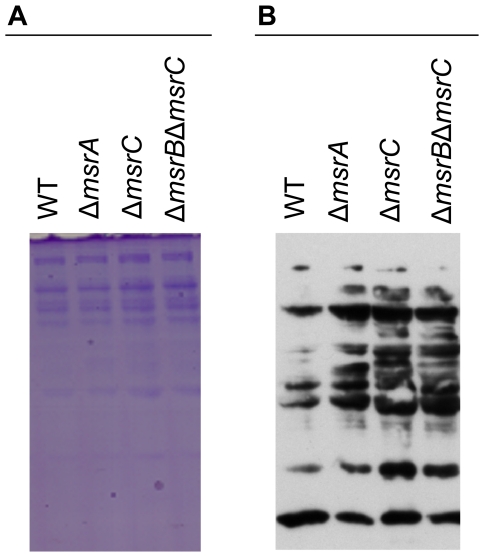
Oxyblot of whole cell extracts of *S*. Typhimurium wild type, Δ*msrA*, Δ*msrC*, Δ*msrB*Δ*msrC*. Cells grown to OD_600_ 1 were incubated in 0.75 mM H_2_O_2_ for 3 h. **A.** SDS-PAGE stained with Coomassie. **B.** Oxyblot film exposed for 2 min.

To clarify the role of MsrC in oxidative stress response we generated mutants with a single deletion in *msrC* and a double mutant with deletions in both genes *msrC* and *msrB* in the *S.* Typhimurium wild type background. The mutants did not show a general growth defect under normal aerobic culture conditions in LB broth (Fig. S2). In the presence of 2 mM exogenous H_2_O_2_ the Δ*msrC* mutant was more susceptible than the wild type after incubation of 6 and 9 hours (Fig. 5). The wild type phenotype could be restored by expressing *msrC* on a plasmid under control of its own promoter. Interestingly, the survival of the Δ*msrB*Δ*msrC* double mutant was more reduced than the Δ*msrC* single mutant in the presence of 2 mM H_2_O_2_ *in vitro* after 6 and 9 hours (Fig. 5) of incubation. Since the Δ*msrA*, Δ*msrC*, and Δ*msrB*Δ*msrC* mutants are more susceptible to H_2_O_2_ *in vitro*, we sought to characterize the oxidized protein profiles of these mutants in comparison to the wild type following exposure to H_2_O_2_. Accumulation of oxidized proteins was visualized using the oxyblot method. Signals of immunoblots of the mutant strains clearly differed from those of the wild type, indicating a higher level of oxidation in the absence of *msrA*, *msrC*, or *msrB* and *msrC* (Fig. 6B).

### Growth of a Δ*msrC* mutant inside activated macrophages

**Figure 7:**
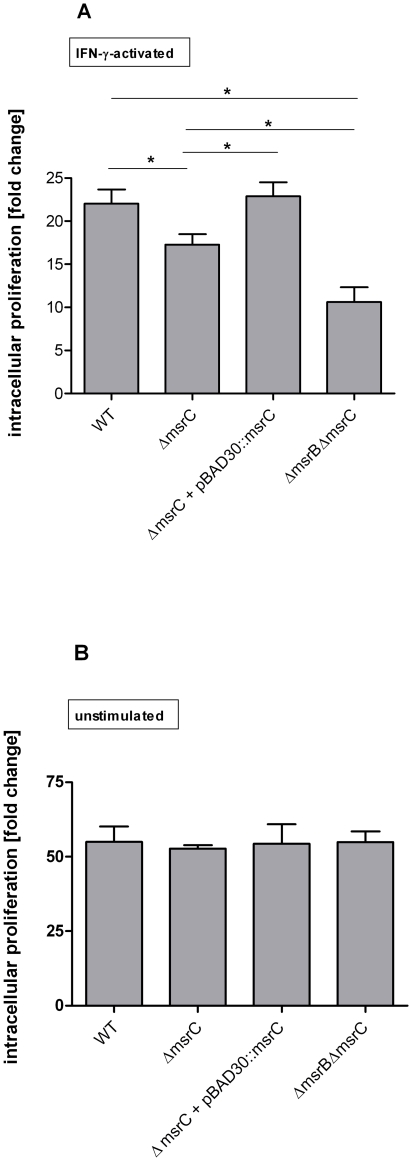
Intracellular proliferation of *S.* Typhimurium wild type, Δ*msrC*, Δ*msrB* Δ***msrC*** **in RAW 264.7 cells.** *S.* Typhimurium wild type, Δ*msrC*, Δ*msrC* + pBAD30::*msrC* and Δ*msrB*Δ*msrC* were tested **A.** in IFN-γ-activated, and **B.** in non-stimulated RAW 264.7 cells. Intracellular proliferation was determined in a gentamicin protection assay. Number of bacteria 16 h post infection was divided by the number of bacteria 2 h post infection. Results shown are the means ± standard error of the mean for three independent experiments, each in triplicate. *P-*values <0.05 were considered to be significant as indicated by asterisks.

To elucidate the role of MsrC during intracellular growth of *S*. Typhimurium, RAW 264.7 macrophages were infected with wild type and mutant strains. The experiment was performed as described above. In IFN-γ-activated RAW 264.7 cells the single Δ*msrC* mutant was attenuated compared to the wild type. The growth of the Δ*msrB*Δ*msrC* mutant was significantly more affected than the Δ*msrC* mutant (Fig. 7A), which is in line with the higher susceptibility of the Δ*msrB*Δ*msrC* double mutant compared to the Δ*msrC* single mutant towards exogenous H_2_O_2_. When we tested the strains in unstimulated RAW 264.7 cells, we found that the mutants were not affected in their intracellular growth in comparison to the wild type (Fig. 7,B).

### *In vivo* susceptibility of a Δ*msrC* mutant in Balb/cJ mice

To test whether MsrC or MsrB and MsrC together affect virulence of *S.* Typhimurium in mice, the fitness of the Δ*msrC* single mutant and the Δ*msrB*Δ*msrC* double mutant were directly compared to that of the wild type in a competition experiment [26]. The experiments were performed as described above. The Δ*msrC* mutant was not attenuated in the liver and in the spleen 1 and 3 days after infection (Table 3 and 4). The Δ*msrB*Δ*msrC* mutant was attenuated in the mouse model of infection in the liver and in the spleen 3 days after infection (Table 4). These data are consistent with the higher susceptibility of the Δ*msrB*Δ*msrC* double mutants compared to the Δ*msrC* mutant towards exogenous H_2_O_2_ and the more severe attenuation of the Δ*msrB*Δ*msrC* double mutant compared to the Δ*msrC* mutant in IFN-γ-activated RAW 264.7 cells. Taken together these results suggest that beside the repair of protein bound MetSO by MsrA and MsrB, the repair of free MetSO is also crucial for resistance of *S*. Typhimurium towards oxidative stress *in vitro* and *in vivo*.

**Table 3:** Competitive indices (CI) of Δ*msrC* and Δ*msrB*Δ*msrC* versus wild type on day 1 post infection.

Strains	CI liver 1 day p.i.	*P*-value*	CI spleen 1 day p.i.	*P*-value*
Δ*msrC* vs. WT	0.91±0.21	>0.05	1.07±0.25	>0.05
Δ*msrB*Δ*msrC* vs. WT	0.67±0.15	<0.05	0.95±0.46	>0.05

**Table 4:** Competitive indices (CI) of Δ*msrC* and Δ*msrB*Δ*msrC* versus wild type on day 3 post infection.

Strains	CI liver 3 days p.i.	*P*-value*	CI spleen 3 days p.i.	*P*-value*
Δ*msrC* vs. WT	0.77±0.13	>0.05	0.82±0.31	>0.05
Δ*msrB*Δ*msrC* vs. WT	0.57±0.15	<0.05	0.46±0.06	<0.05

*P-values were calculated using the Wilcoxon Signed Rank Test to test whether the actual median differs significantly from 1 (theoretical median). CIs are shown as medians ± standard deviation.

## Discussion

Previously MsrA and MsrB were identified as the two principle methionine sulfoxide reductases that play a role in protecting bacteria against oxidative damage [28]. The genetic organization of *msrA* and *msrB* varies between organisms [29], [30]. In *S.* Typhimurium and other pathogens such as *E. coli, C. jejuni, S. aureus, E. faecalis*, *M. smegmatis* and *M. tuberculosis msrA* and *msrB* are transcribed independently as single genes [13]–[17], [20], [31]–[33]. Of these, only *S*. Typhimurium and *M. tuberculosis* are considered facultative intracellular pathogens, and they are able to replicate inside macrophages in the host. In *M. tuberculosis* deletion of *msrA* did not show a phenotype when challenged with exogenous H_2_O_2_, in activated macrophages, or in mice. Thus of the two intracellular pathogens in which the *msr*-system has been analyzed in detail, only in *Salmonella* MsrA is highly efficient in mediating resistance to oxidative stress.

In contrast to the Δ*msrA* mutant, the Δ*msrB* single mutant of *S.* Typhimurium showed the wild type phenotype at all conditions tested. Our results further showed that recombinant MsrA reduced both free and peptidyl Met-*S*-SO, whereas MsrB was specific only for peptidyl Met-*R*-SO. In this respect, our *in vitro* data concur with the substrate specificities of MsrA and MsrB in *M. tuberculosis.* In *M. tuberculosis* the purified MsrA is active against both free and peptidyl Met-*S*-SO, whereas MsrB showed weak activity for peptidyl Met-*R*-SO, and was inactive towards free Met-*R*-SO [20].

We speculated that an explanation for the phenotypic difference of the Δ*msrA* and Δ*msrB* mutant of *S*. Typhimurium during oxidative stress could be the limited substrate range of MsrB. In contrast to MsrA which is active against both peptidyl and free Met-*S*-SO; MsrB is only active against the peptidyl Met-*R*-SO. This idea implied that yet another methionine sulfoxide reductase for the *R*-form exists that could play a role in oxidative stress response of *S.* Typhimurium. A free methionine-*R*-sulfoxide reductase (MsrC) was first characterized by Lin and colleagues in *E. coli* [10], and was subsequently purified from *N. meningitidis* and from *S. aureus*. High substrate specificity for free Met-*R*-SO was reported in all three bacterial organisms, and detailed analysis with respect to biochemical properties and structural composition was carried out [10], [21], [22]. Lee and Gladyshev described MsrC as a highly specific Msr for free Met-*R*-SO that does not reduce peptidyl methionine sulfoxides, and compensates for the low or missing activity of MsrB with respect to free Met-*R*-SO [34].

However, none of the studies performed experiments to elucidate the role of MsrC in bacterial pathogenesis. In *S. cerevisiae* the role of MsrC in reducing free Met-*R*-SO was also clearly demonstrated [11]. Here the authors showed that MsrC mediates resistance to *S. cerevisiae* when challenged with H_2_O_2_ *in vitro* [11]. The influence of MsrC for growth or survival inside macrophages or in mice could not be studied in *S. cerevisae*, as the organism is non-pathogenic. The present work demonstrates that the methionine sulfoxide reductase MsrC is essential for growth and survival of an intracellular pathogen in macrophages, and, in combination with MsrB, in mice. We also showed by growth experiments with a *msrC* deficient mutant in a methionine auxotrophic background that in *S.* Typhimurium MsrC repairs free Met-*R*-SO. In all biochemical studies available at present, MsrC has been shown to be specific for free Met-*R*-SO, and there is no experimental evidence that MsrC can use peptidyl Met-*R*-SO as a substrate. In addition, we performed computational analysis and homology modelling using the available structural information of MsrC from *E. coli* (pdb: 1vhm) [10], and predicted the binding of free Met-*R*-SO but not protein based Met-*R*-SO as substrates for MsrC due to the conformation of its substrate binding pocket (data not shown).

It has been suggested that the cycle of oxidation of methionine and enzymatic reduction serves as a sink for oxidants [8]. Within this cycle MsrA, MsrB and MsrC could act together, to completely reduce free and protein based methionine sulfoxides [34]. Our data showed that MsrB has a function in reduction of protein bound Met-*R-*SO. The lack of activity towards free Met-*R*-SO can be compensated by MsrC [34]. Thus in contrast to Met-*S*-SO the repair of Met-*R*-SO requires two enzymes, one that is specific for the free amino acid and another for the protein-based form. In the Δ*msrC* mutant, Met-*R*-SO within proteins can still be reduced by MsrB explaining its moderate phenotype. The synergistic effect of the *msrB* and *msrC* double mutation in *S.* Typhimurium following exposure to H_2_O_2_, in activated RAW 264.7 macrophages, and in mice, is due to the inability of the mutant to repair both free Met-*R*-SO and peptidyl Met-*R*-SO, causing a more dramatic decline of the overall scavenger capacity of methionine in the cell. It is noteworthy that a Δ*msrA* mutant, which typically acts on both free and peptidyl-Met-*S*-SO shows a strong phenotype in *Salmonella* (this study) and in most other bacteria [13], [16], [19], [32], [35]. Thus free methionine plays a more important role during oxidative stress in bacteria than predicted so far.

Finally MsrC has been proposed to act as a signaling molecule in oxidative stress response as the protein contains a GAF domain [10], [22], and it is tempting to speculate that alteration of signaling contributes to the phenotype of the Δ*msrC* mutant in *S.* Typhimurium. The GAF domain within the MsrC molecule was first described by Lin and coworkers, who also solved the structure of the protein. Based on the identification of the GAF domain it was speculated that the binding and reduction of free methionine sulfoxide could mediate cell signaling in response to oxidative stress [10]. An alternative hypothesis for the role of the GAF domain, however, challenged the idea of MsrC being a signaling molecule, and suggested that MsrC is an unusual example for a GAF fold that simply acts as an enzyme without a modular function [22].

Thus in summary, we showed that mutants of *S.* Typhimurium that lack components of the methionine sulfoxide reductase pathway are attenuated *in vitro* when exposed to H_2_O_2_, inside activated macrophages and in mice. Previously, MsrA and MsrB were considered to be the principle enzymes of the *msr*-system that play a role in bacterial resistance to oxidative stress. Here we showed that in addition MsrC contributes significantly to counteract the damage that is afflicted to bacterial pathogens by oxidative stress.

## Materials and Methods

### Ethics Statement

The mouse experiments were approved by Stockholm Norra Djurförsöksetiska Nämnd, Stockholms Tingsrätt, Box 8307, 104 20 Stockholm. The mouse experiments were performed in the animal facility at the Department of Microbiology, Tumor and Cell Biology, Karolinska Institutet (Stockholm, Sweden) in accordance with both institutional and national guidelines (ethical permit N345/08).

### Bacterial strains and plasmids

Bacterial strains used in this study are shown in Table 5. *Salmonella enterica* serovar Typhimurium ATCC14028 (*S.* Typhimurium) was used as wild type and all mutants were generated in this genetic background. *Salmonella* was grown overnight in LB broth in a “roller drum” under aerobic conditions at 37°C with antibiotics if necessary (ampicillin 100 µg ml^−1^, chloramphenicol 10 µg ml^−1^, kanamycin 25 µg ml^−1^). *E. coli* was grown under shaking conditions at 37°C in LB medium with appropriate antibiotics.

**Table 5:** Strains used in this study.

Strain	characteristics	selective marker	reference
*E. coli* HB101	F–, *thi*-1, *hsd*S20 (r_B_^–^, m_B_^–^), *sup*E44, *rec*A13, *ara*-14, *leu*B6, *pro*A2, *lac*Y1, *gal*K2, *rps*L20 (str^r^), *xyl*-5, *mtl*-1.		Promega
T7 *lysY* Express	MiniF, *lysY* (Cam^R^) */ fhuA2 lacZ::T7 gene1 [lon] ompT gal sulA11 R(mcr-73::miniTn10--*Tet^S^ *)2 [dcm] R(zgb-210::Tn10--*Tet^S^ *) endA1 Δ(mcrC-mrr)114::IS10*	chloramphenicol	NEB
VK1	*E. coli* T7 *lysY* Express transformed with pET28c::*msrA*	kanamycin	This study
VK2	*E. coli* T7 *lysY* Express transformed with pET28c::*msrB*	kanamycin	This study
*S*. Typhimurium	wild type	-	ATCC 14028
SHO11	*ΔmsrA*	chloramphenicol	This study
SHO18	*ΔmsrAΔmsrB*	chloramphenicol, kanamycin	This study
SHO19	*ΔmsrB*	kanamycin	This study
SHO35	*ΔmsrA* transformed with pBAD30::*msrA*	chloramphenicol, ampicillin	This study
SHO36	*ΔmsrAΔmsrB* transformed with pBAD30::*msrA*	chloramphenicol, kanamycin, ampicillin	This study
LD24	*ΔmetCΔmsrB*	kanamycin	This study
LD40	*ΔmetCΔmsrC*	chloramphenicol	This study
LD41	*ΔmetCΔmsrBΔmsrC*	chloramphenicol, kanamycin	This study
LD39	*ΔmsrC*	chloramphenicol	This study
LD49	*ΔmsrBΔmsrC*	chloramphenicol	This study
LD55	*ΔmsrC* transformed with pBAD30::*msrC*	chloramphenicol, ampicillin	This study

### Construction of deletion mutants in *S.* Typhimurium 14028 and complementation

DNA sequences of *S*. Typhimurium were obtained from the database *coli*BASE (strain LT2). Generation of deletion mutants for the open reading frames *msrA* (STM4408), *msrB* (STM1291), *metC* (STM3161) and *msrC* (STM1847) was performed using the one-step inactivation via homologous recombination [36]. Primers and plasmids are listed in Table 6 and 7.

**Table 6:** Primers used in this study.

Primer	Sequence (5′→3′)	Purpose
msrA1	ATG CGT TAC CAG GAC GCA ACA CCC CGA TGC GAT CGC CAC TCT GCG TAG GCT GGA GCT GCT TC	Deletion of *msrA*
msrA2	GCG ATG GTC TCC GGC GGC GGT CAT TGC CGA CTG AAA GCG CTC GCG TAT GAA TAT CCT CCT TAG	Deletion of *msrA*
msrA ST fwd	CTGGTTACTCAAGCAGATGCG	Confirmation of m*srA* deletion
msrA ST rvs	CAGGGCTAGTATAGCGTAAGC	Confirmation of m*srA* deletion
msrB1	GTG TTA ATG TTT TGT TAG AAT CGG TCA GGC AAT GTG AGC ACG GTA GGC TGG AGC TGC TTC	Deletion of *msrB*
msrB2	GTC CGC TCC TGT GGA ATA ATT TGC TGA ATC GTT TTT TCA GCC TAT GAA TAT CCT CCT TAG	Deletion of *msrB*
msrB test 1	GATTCGTGAGCCGCCATTTC	Confirmation of *msrB* deletion
msrB test 2	GATACACTTCCGGCGTCATG	Confirmation of *msrB* deletion
metC_For	CAT GCT AGT TTA GAC ATC CAG ACG GTTAAA ATC AGG AAA CGC AAC GTA GGC TGG AGC TGC TTC	Deletion of *metC*
metC_Rev	CAG ACT TTT CCA CGG AAA TTG TCT GCA TAT ATG TCC ATC CCC GCC TAT GAA TAT CCT CCT TAG	Deletion of *metC*
k metC_For	CGT CGC CAG GGT GCA GAT GG	Confirmation of *metC* deletion
k metC_Rev	GCC TTG ATC CCG GAC GCA AC	Confirmation of *metC* deletion
msrC_For	GAC CAC GAA GTG ATT ATA TAA TGA GCA AAA CAG AAC TAT ACG CGG GTA GGC TGG AGC TGC TTC	Deletion *msrC*
msrC_Rev	CAA ACG CCG TGC TAT CTA TAT CCA GCA CGC CGA TAA TCC GTT CGC TAT GAA TAT CCT CCT TAG	Deletion *msrC*
k msrC_For	GAG CAA GAA CGC ATT TAA TGC	Confirmation of *msrC* deletion
k msrC_Rev	GCG TAC GCA GGC CGT GT TC	Confirmation of *msrC* deletion
msrA1_HindIII	ATA TAT AAG CTT CTC AGG CAC AGT AAG CTA AC	Complementation with *msrA*
msrA2_HindIII	ATA TAT AAG CTT TTC AAC TCC GAC AAG TTC CC	Complementation with *msrA*
msrC_ HindIII	ATA TAT AAG CTT CTG CGT CAT TTG CCA GAT GC	Complementation with *msrC*
msrC_ HindIII	ATA TAT AAG CTT TCG GCC AGA AAC GCG ATA AC	Complementation with *msrC*
msrA_F_NheI	ATA TAT GCT AGC ATG AGT TTA TTT GAT AAA AAA CAT CTG	Overexpression MsrA
msrA_R_BamHI	ATA TAT GGA TCC TCA CGC GTC AGG CGG CAG G	Overexpression MsrA
msrB_F_NheI	ATA TAT GCT AGC GTG AGC ACG TTT AAA GTG AGA TG	Overexpression MsrB
msrB_R_BamHI	ATA TAT GGA TCC TCA GCC TTT CAG TTG ATC GCC G	Overexpression MsrB

**Table 7:** Plasmids used in this study.

plasmid	selection marker	source
pKD3	chloramphenicol, ampicillin	[8]
pKD4	kanamycin, ampicillin	[8]
pKD46	ampicillin	[8]
pCP20	chloramphenicol, ampicillin	[8]
pBAD30	ampicillin	[16]
pBAD30::*msrA*	ampicillin	this study
pBAD30::*msrC*	ampicillin	this study
pET28c	kanamycin	Novagen
pET28c::*msrA*	kanamycin	this study
pET28c::*msrB*	kanamycin	this study

Briefly, primers were designed that consist of a 42 – 45 bp region homologous to the flanking regions of the gene to be deleted, and an 18 bp region homologous to the resistance cassette of pKD3 or pKD4, respectively (Table 6). PCR products contain a FRT-flanked chloramphenicol (pKD3) or kanamycin (pKD4) resistance cassette flanked by 42 – 45 bp regions homologous to the adjacent regions of the gene to be deleted.

The helper plasmid pKD46 (Table 7) carrying the Red Recombinase under the control of an arabinose inducible P*_araB_* promotor was transformed into an electrocompetent *S.* Typhimurium receiver strain. After electroporation transformants were selected at 30°C on LB agar plates with ampicillin. Next step the pKD46 carrying clone was cultivated in 50 ml LB broth with ampicillin and 20 mM L-arabinose for induction of the Red Recombinase to OD_600_ 0.3 - 0.4. Cells were prepared for electroporation and purified PCR products specific for deletion of *msrA*, *msrB*, *metC* or *msrC*, respectively, were transformed into the receiver strain. Site-specific recombination mediated by the Red recombinase was carried out during 3 h of incubation after electroporation without shaking. 100 µl of the bacteria suspension were plated on LB agar with the appropriate antibiotic and incubated over night at 37°C. Chloramphenicol or kanamycin resistant clones were selected and subcultured twice in 5 ml LB at 42°C to get rid off the temperature sensitive helper plasmid pKD46. Obtained mutants were confirmed by PCR using primers that bind in the flanking regions of the disrupted gene (Table 6).

When necessary, antibiotic resistance genes were eliminated by the temperature-sensitive plasmid pCP20 (Table 7) carrying a FLP recombinase. Mutants containing FRT-flanked antibiotic resistance cassettes were transformed with pCP20 and ampicillin-resistant transformants were selected at 30°C. To get rid off the temperature sensitive helper plasmid pCP20 the mutants were cultivated at 42°C.

The construction of the double Δ*msrA*Δ*msrB* deletion mutant was performed by using the single *msrA* mutant as background strain. Mutations were transferred between different strains by transduction with the bacteriophage P22*int* [37] to construct the multiple deletion mutants Δ*metC*Δ*msrB* and *ΔmetC*Δ*msrC*, Δ*metC*Δ*msrB*Δ*msrC* and Δ*msrB*Δ*msrC* .

For complementation of the deletion mutants with wild type genes from *S.* Typhimurium 14028 the open reading frames *msrA* and *msrC* including upstream regions between 250bp – 400 bp were amplified by PCR using Phusion™ High Fidelity DNA Polymerase (NEB). Primers (Table 6) included restriction sites for subsequent cloning into the vector pBAD30 [38]. All genes were under control of their own promoter.

### Growth of *S*. Typhimurium in LB medium under aerobic conditions

Over night cultures of *S*. Typhimurium were diluted to an OD_600_ of 0.02 and were then incubated aerobically under shaking conditions in LB medium (37°C, 130 rpm). The OD_600_ was measured every 45 min. Experiments were done in duplicates.

### Susceptibility of *S.* Typhimurium to oxidative stress *in vitro*

Hydrogen peroxide was purchased from Sigma. Over night cultures of *S.* Typhimurium were diluted to an OD_600_ of 0.02 and 100 µl diluted culture were mixed with 100 µl of hydrogen peroxide (4 mM) at a final concentration of 2 mM. The assay was carried out in LB broth and the 96-well-plates were incubated at 37°C without shaking. Killing of bacteria was determined by plating for colony forming units on LB agar after 3, 6 and 9 h of exposure. Experiments were done in duplicates.

For experiments using the OxyBlot™ protein oxidation detection kit (Millipore), *S.* Typhimurium strains were grown in 25 ml LB to OD_600_ 1. Cultures of *S.* Typhimurium were exposed to H_2_O_2_ at a final concentration of 0.75 mM and incubated for 3 hours at 37°C without shaking. Cells were centrifuged at 9273×g, dissolved in 500 µl 10 mM Tris, 25 mM NaCl and 50 mM DTT pH 7.5 and transferred to Lysing Matrix B tubes (MP Biomedicals). Cells were disrupted mechanically using the Hybaid Ribolyser for 40 seconds at speed 6.0. After cooling down the samples on ice cell debris were removed 3 min at 16000×*g*, the supernatant was transferred into a new reaction tube.

Detection of whole cell protein profiles using the OxyBlot™ protein oxidation detection kit (Millipore) was performed according to the manual provided by the manufacturer. Briefly, 10 µg protein (5 µl) from each culture supernatant was mixed with 5 µl of 12% SDS, subsequently mixed with 10 µl of the derivatizing agent 1×DNPH (dinitrophenyl hydrazine) and incubated at room temperature for 15 min. Reaction was stopped by adding 7.5 µl neutralization solution to all tubes, and samples were loaded on a 12.5% SDS-PAGE.

The proteins on the SDS-PAGE were electrotransferred onto a nitrocellulose membrane (Whatman Protran nitrocellulose), and subsequently blocked for 1 h in blocking buffer containing PBS, 1% BSA and 0.05% Tween 20. The membrane was incubated in rabbit anti-DNP-antibody (1∶150) diluted in TBS with 1% BSA over night at 4°C, and subsequently in goat anti-rabbit IgG horseradish peroxidase conjugate (1∶300) diluted in TBS with 1% BSA for 1 h. Oxidized proteins were visualized using the ECL substrate (SuperSignal West Pico Chemiluminescent Substrate, Thermo Scientific) according to manufacturer's directions.

### Infection of the macrophage cell line RAW 264.7

RAW 264.7 macrophages (ECACC) were grown in RPMI (Gibco) supplemented with 10% heat inactivated FCS (Sigma), 4 mM Glutamine (Gibco) and 20 mM Hepes (Gibco). Infection experiments were performed as described earlier [39]. Briefly, cells were seeded in 24-well plates at a density of 5×10^4^ cells per well. Over night cultures of *S.* Typhimurium were diluted to an OD_600_ of 0.2 and 1.5 ml of bacteria were harvested, washed twice with PBS, and resuspended in 1 ml RPMI medium without any supplements. The bacterial suspension was further diluted 1∶100 in RPMI and 100 µl were used for infection of RAW 264.7 cells in 900 µl (MOI of 10∶1) RPMI medium without any supplements. Plates were centrifuged at 220×g for 5 min and incubated 30 min at 37°C and 5% CO_2_. Extracellular bacteria were removed by washing three times with PBS and incubated 60 min with RPMI containing 100 µg ml^−1^ gentamicin. Medium was replaced by RPMI with 10 µg ml^−1^ gentamicin for the rest of the experiment. To assess the bacterial load in the macrophages 2 h and 16 h post infection, cells were washed three times with PBS and lysed with 1 ml sterile bidistilled water. Bacteria were plated on LB agar for colony forming units (cfu). To calculate bacterial growth, cfu count 16 h post infection was divided by the cfu count 2 h post infection. For activation, macrophages were stimulated with murine IFN-γ (Sigma) 18–24 h prior infection. All experiments were performed in triplicates.

### Competitive infection of Balb/cJ mice with *S*. Typhimurium

Fitness of mutant strains was compared to wild type by a competition experiment in mice. Strains were taken from an over night LB agar plate, resuspended in PBS and the number of cells was adjusted to 5×10^3^ bacteria for each strain. For infection a 1∶1 mixture of two strains with a total of 1×10^4^ bacteria in 100 µl sterile PBS was used for infection. For each time point and mutant groups of 5 female BALB/cJ mice at the age of 6 to 8 weeks were infected intraperitoneally. Mice were sacrificed on day 1 and day 3 post infection and the livers and spleens were removed and homogenized in PBS. Bacteria were plated for cfu on LB agar and on LB agar containing the appropriate antibiotic (chloramphenicol for Δ*msrA*, Δ*msrA*Δ*msrB*, Δ*msrC*, Δ*msrB*Δ*msrC*, kanamycin for Δ*msrB*) to distinguish between wild type and mutant. Competitive indices (CI) were calculated as described previously [26]. Briefly, the CI is the ratio between the mutant and the wild type at day 1 or 3 divided by the ratio of mutant and wild type at day 0. The competitive index measures the attenuation of the tested mutant strain.

### Culture of *Salmonella* on methionine and methionine sulfoxide

Overnight cultures of *S.* Typhimurium in LB were harvested by centrifugation, washed three times in M9 minimal medium, and inoculated in M9 minimal medium at an OD_600_ 0.05. 13 µg ml^−1^ of methionine or Met-*R*-SO, respectively, were added as methionine source. The cells were incubated aerobically under shaking conditions (37°C, 130 rpm). Growth was measured by OD_600_ every hour for 9 h of cultivation. Experiments were done in triplicates.

### Overexpression and purification of S. Typhimurium His-tagged MsrA and MsrB

The open reading frames of *msrA* (STM4408) and *msrB* (STM1291) from *S.* Typhimurium were amplified from genomic DNA by PCR. Primers (Table 6) included restriction sites for subsequent cloning into the T7 overexpression vector pET28c (Novagen, Table 7) according to manufacturer's instructions. Plasmids, designated pET28c::*msrA* and pET28c::*msrB* (Table 7), respectively, were transformed into *E. coli* T7 *lysY* Express (NEB) for expression of the recombinant His-tagged proteins. Expression of MsrA and MsrB was induced by addition of 0.5 mM IPTG at an OD_600_ ∼0,8, and cells were grown for another 5 h. Bacteria were harvested by centrifugation (3000×g, 25 min, 4°C) and resuspended in binding buffer containing 50 mM NaH_2_PO_4_, 300 mM NaCl and 10 mM imidazole (pH 8.0 for MsrA, pH 8.5 for MsrB). Cells were lysed by gently shaking with lysozyme (0.3 mg ml^−1^, Roth) for 1 h at 4°C, and by sonication in a SONOPLUS sonicator (Bandelin). The supernatant was purified using a Ni-NTA column (QIAGEN) and the Bio Logic Duo Flow System (Biorad). Buffer used for pre-equilibration of the Ni-NTA column and washing steps (binding buffer, described above) contained 20 mM imidazole, buffer used for elution contained 500 mM imidazol. The His-tagged proteins were eluted by applying a linear gradient from 0 to 100% elution buffer to the column. Fractions with MsrA or MsrB were pooled and desalted using size-exclusion chromatography into 10 mM Tris and 1 mM EDTA (pH 8.0 for MsrA, pH 8.5 for MsrB). Proteins were concentrated by centrifugation (2500×g, 4°C) using VIVASPIN 20 concentrators (Sartorius Stedim Biotech). Protein concentrations of MsrA and MsrB were determined by the Quick Start Bradford Protein Assay (Bio-Rad).

### NADPH linked reductase activity assay

MsrA and MsrB activities were measured by monitoring NADPH oxidation at 340 nm by means of a coupled test system. Thioredoxin B (TrxB) and thioredoxin reductase (TrxR) from *M. tuberculosis* were used to regenerate MsrA and MsrB to its reduced form. All enzyme assays were performed at 25°C in a final volume of 500 µl, using an UVmc2 spectrophotometer (Safas S.A.). The reaction mixture contained 10 µM purified MsrA or MsrB, 10 µM TrxR, 10 µM TrxB, 450 µM NADPH, and 5 µM to 500 µM of (*R*) or (*S*) methyl *p*-tolyl sulfoxide (Sigma) or (*R*) or (*S*) N-acetyl methionine sulfoxide (MOLISA), respectively. After 10 min pre-incubation reactions were started by the addition of the substrate.

### Acetylation of Met-*S*-SO or Met-*R*-SO

Met-*S*-SO or Met-*R*-SO (102 mg, 0.62 mmol) and NaHCO_3_ (130 mg, 1.54 mmol; 2.5 eq) were dissolved in ice-cold water (1.5 ml). The solution was treated with acetic anhydride (88 µl, 0.93 mmol; 1.5 eq) and stirred vigorously for 3 hours at room temperature. It was then adjusted to pH ∼3 with 1 M KHSO_4_-solution, and diluted with MeOH, transferred to a 100 ml-flask, and evaporated to dryness *in vacuo*. Silica gel and CH_2_Cl_2_/MeOH (1∶1) were added, and the mixture was again evaporated *in vacuo* to yield a dry powder which was poured onto a pre-filled chromatography column. Regular flash chromatography (CH_2_Cl_2_/MeOH = 3∶1+1% HOAc) and co-evaporation with toluene *in vacuo* yielded the product as colorless glass (58–75 mg; 45–58%).

We used ^1^H-NMR spectrum to confirm the structure of N-acetyl Met-*S*-SO and of N-acetyl Met-*R*-SO, respectively:

N-acetyl Met-*S*-SO: ^1^H-NMR (600 MHz, MeOH-d_4_), *δ* = 4.52 (dd, 8.4 Hz, 5.3 Hz, 1 H; C2-H), 2.94-2.83 (m, 2 H; C4-H_2_), 2.65 (s, 3 H; C6-H_3_), 2.36–2.28 (m, 1 H, C3-H'), 2.14–2.06 (m, 1 H, C3-H″), 2.00 (s, 3 H; N-CO-CH_3_) ppm

N-acetyl Met-*R*-SO: ^1^H-NMR (600 MHz, MeOH-d_4_), *δ = *4.50 Δ(dd, 8.7 Hz, 4.8 Hz, 1 H; C2-H), 2.94 (ddd, 13.2 Hz, 10.0 Hz, 6.4 Hz, 1 H; C4-H'), 2.80 (ddd, 13.2 Hz, 10.0 Hz, 4.9 Hz, 1 H; C4-H″), 2.65 (s, 3 H; C6-H_3_), 2.35–2.28 (m, 1 H, C3-H'), 2.14–2.07 (m, 1 H, C3-H″), 2.00 (s, 3 H; N-CO-CH_3_) ppm.

### Statistical analysis

The *t-*test was applied for statistical analyses and *P-*values <0.05 were considered to be significant. Significance of the competitive indices was calculated using the Wilcoxon Signed Rank Test.

## Supporting Information

Figure S1 **Growth curve of** ***S*** **. Typhimurium wild type and mutants in LB medium.** Over night cultures of *S*. Typhimurium were diluted to an OD_600_ of 0.02 and growth of all strains was measured every 45 min.
(TIF)

Figure S2 **Growth curve of** ***S*** **. Typhimurium wild type and mutants in LB medium.** Over night cultures of *S*. Typhimurium were diluted to an OD_600_ of 0.02 and growth of all strains was measured every 45 min.
(TIF)
